# Lady Roberts' Field Glass Fund

**Published:** 1918-12-28

**Authors:** 

**Affiliations:** 64 Victoria Street, Westminster, S.W. 1.


### The Editor's Letter-Box.

(*Continued from page* 273.)

### Lady Roberts' Field Glass Fund.

#### Return of Glasses now Asked For.

*To the Editor of* The Hospital.

Sir,—May I, through the hospitality of your columns, ask all officers and others who have received glasses or telescopes on loan through my Fund to send them back to me now for return to their owners. All instruments lent through my Fund bear the letters N.S.L. (National Service League) followed by a letter and a number. I should be glad if officers and others returning glasses would enclose in the case a note of acknowledgment for the owner.

I wish to record my gratitude not only to the public for the munificent loan of 30,000 glasses, but also to the Press for the valued help which it has given this undertaking.

The address for glasses and correspondence is The Manager, Lady Roberts' Field Glass Fund, 64 Victoria Street, S.W. 1.—Yours faithfully, Roberts.

64 Victoria Street,
Westminster, S.W. 1.

